# A Case of Intrathoracic Stomach and Spleen After Aortic Repair: An Unusual Complication

**DOI:** 10.4021/jocmr2010.04.293w

**Published:** 2010-06-15

**Authors:** Stephanie A.M. de Haas, P.K.C. van der Weijden, P. Steenvoorde, P.P. Hedeman Joosten

**Affiliations:** a Department of Intensive Care, Rijnland Hospital Leiderdorp, The Netherlands; b Department of Surgery, Rijnland Hospital Leiderdorp, The Netherlands

## Abstract

A patient is described who presented with an intrathoracic stomach and spleen two weeks after aortic repair for an aortoduodenal fistula. At an urgent laparotomy the stomach was repositioned and the spleen removed. The patient recovered fully. The possible mechanism of this severe complication is discussed and relevant literature reviewed.

**Keywords:**

Aortoduodenal fistula; Complication; Intrathoracic stomach; Intrathoracic spleen

## Introduction

Aorta repair in abdominal aortic aneurysms is a common procedure and is performed approximately 2000 times a year in the Netherlands. Elective treatment is recommended when AAA size reaches 5.5 cm in diameter due to higher rates of rupture [1].

The 30-day mortality rate is 5% after operation [1]. Common complications of this procedure are bleeding after surgery, colon ischemia, ileus, and other postoperative complications such as pneumonia (2%), septic shock (2%) and renal failure (4.2%). Late complications are aortoenteric fistulas and graft infections (3.8%) [1-3].

In this article a patient is described who presented with acute dyspnea just prior to being discharged from the hospital, after he was successfully operated for an aortoduodenal fistula. Diagnosis of this, uncommon complication, was made on a simple X-ray. Urgent re-laparotomy had to be performed. In this article the patient is described and relevant literature reviewed.

## Case Report

A 57-year-old male was admitted to our hospital with hematemesis. His relevant medical history consisted of hypertension, aorta bifemoral prosthesis for an infrarenal abdominal aneurysm three years ago and a coronary artery bypass graft.

An aortoduodenal fistula was presumed after gastroscopy and CT-examination of the abdomen. At laparotomy the preoperative diagnosis of an aortoduodenal fistula was confirmed. The old prosthesis was replaced by a Rifampicine coated bifurcation prosthesis (Aorta bifurcation prosthesis Silver - Sigma medical, Apeldoorn, the Netherlands).

**Figure 1.**
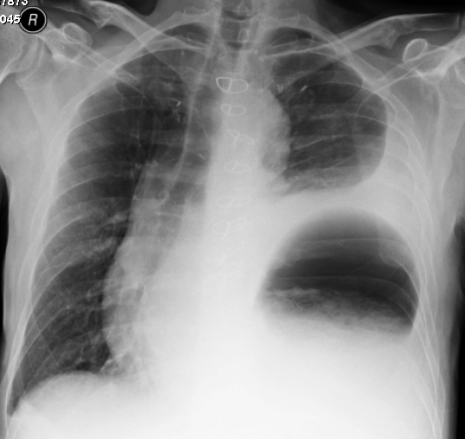
X-ray of the thorax with suspected intrathoracic stomach.

**Figure 2.**
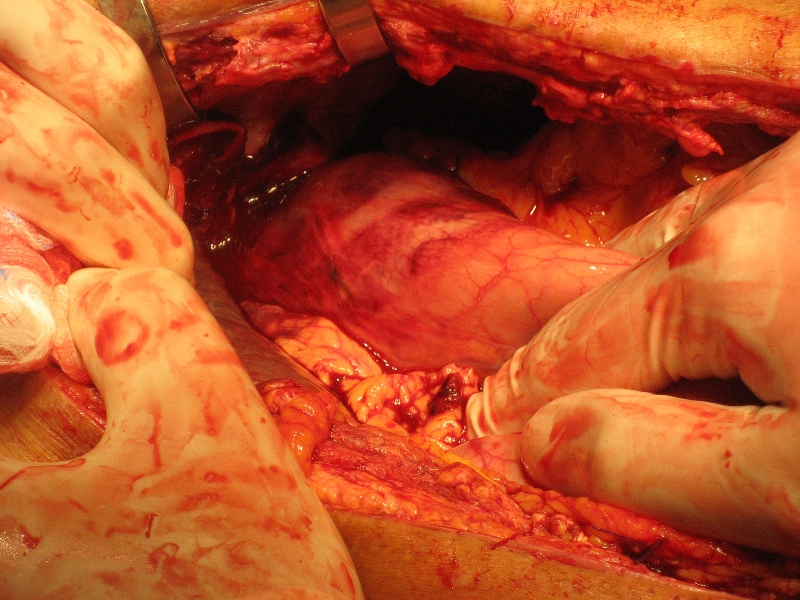
The intrathoracic stomach could be replaced.

**Figure 3.**
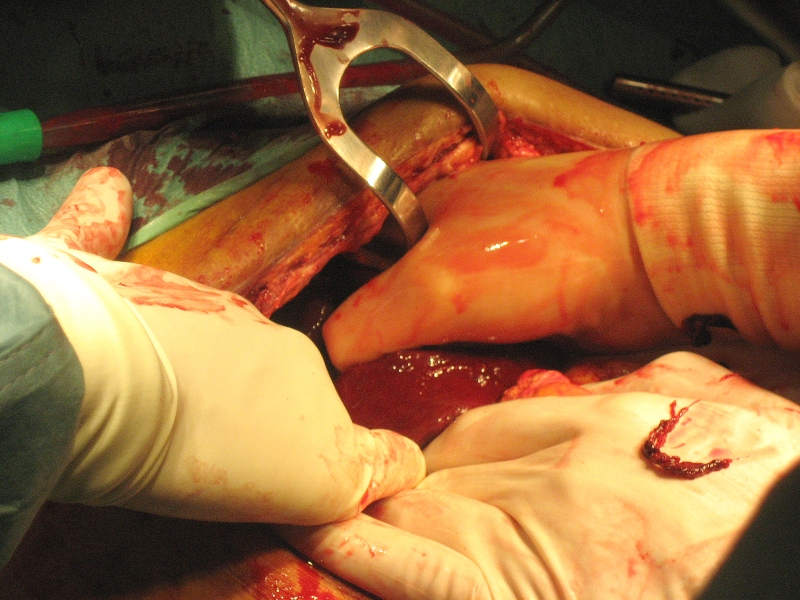
Removal of the decapsulated spleen.

The direct post-operative period was without any complications besides chylus leakage for which a medium chain triglycerides (MCT) diet was started and which resolved fully. However on the 27th post-operative day the patient complained of acute dyspnea and pain on the left thorax and in the left flank. At auscultation decreased breath sounds were heard on the left side. On the X-ray of the thorax an intrathoracic stomach was presumed (Fig. 1). For further diagnosis a CT-scan of the thorax and abdomen was performed which showed fluid collections perisplenic, perihepatic and paracolic: probably caused by a rupture of the spleen with active bleeding. Furthermore an intrathoracic positioned stomach on the left side of the thorax with a mediastinal shift to the right and total atelectasis of the left lower lobe was diagnosed. A re-laparotomy was performed: the spleen and stomach were indeed intrathoracic. The spleen was completely decapsulated and had to be removed (Fig. 2), the stomach could be repositioned (Fig. 3). A defect in the diaphragm was closed with simple Vicryl sutures. Besides a pulmonary embolism the post-operative period was uneventful. The patient could be discharged from the hospital 31 days after the re-laparotomy and is now in a good clinical condition.

## Discussion

Secondary aortoduodenal fistulas are a rare but very important complication of abdominal aortic reconstruction with high morbidity and mortality rates. The complication often occurs months to years after the original surgery.

Herniation of the stomach into the thoracic cavity after surgery is a rare complication. It has been described as a late complication after Nissen fundoplication [4] and after gastric banding for morbid obesity [5].

An intrathoracic spleen is even more rare, it is described in adults after trauma [6]. This is the first case report of a patient with an intrathoracic stomach and spleen after aorta repair. Most probably the stomach had moved into the thoracic cavity through a preexistent sliding hernia seen at gastroscopy.

The spleen was decapsulated by tension caused from the stomach moving into the thoracic cavity. The dyspnea appears to be caused by the mediastinal shift to the right as well as the total atelectasis of the left lung. Urgent laparotomy was necessary for removal of the bleeding decapsulated spleen, and to reposition the stomach with repair of the diaphragm.
